# CD28 Deficiency Ameliorates Blast Exposure-Induced Lung Inflammation, Oxidative Stress, Apoptosis, and T Cell Accumulation in the Lungs via the PI3K/Akt/FoxO1 Signaling Pathway

**DOI:** 10.1155/2019/4848560

**Published:** 2019-09-02

**Authors:** Yunen Liu, Changci Tong, Ying Xu, Peifang Cong, Ying Liu, Lin Shi, Xiuyun Shi, Yan Zhao, Guangyuan Bi, Hongxu Jin, Mingxiao Hou

**Affiliations:** ^1^Emergency Medicine, Department of General Hospital of Northern Theater Command, Laboratory of Rescue Center of Severe Trauma PLA, No. 83 Road, Shenhe District, Shenyang l10016, China; ^2^Radiation Oncology, Department of General Hospital of Northern Theater Command, Shenyang l10016, China; ^3^Institute of Metal Research, Chinese Academy of Sciences, 72 Wenhua Road, Shenyang 110016, China; ^4^Medical Service Department, Joint Service College of National Defense University, PLA 23rd, Taiping Road, Haitian District, Beijing 100858, China

## Abstract

Although CD28 is associated with the expression of inflammatory mediators, apoptosis-related protein, immunosuppression, and tumorigenesis, the effects of CD28 deficiency on blast exposure-induced lung injury have not been investigated. In this study, we have explored the effects of CD28 on blast exposure-induced lung injury and studied its potential molecular mechanisms. A mouse model of blast exposure-induced acute lung injury was established. Sixty C57BL/6 wild-type (WT) and CD28 knockout (CD28^−/−^) mice were randomly divided into control or model groups. Lung tissue samples were collected 24 h and 48 h after blast injury. Histopathological changes and the expressions of inflammatory-related proteins were detected by hematoxylin-eosin, immunohistochemistry, and immunofluorescence staining. Apoptosis and oxidative stress were evaluated by terminal deoxynucleotidyl transferase dUTP nick end labeling (TUNEL) staining and reactive oxygen species (ROS). Inflammation, apoptosis, oxidative stress, and related pathway protein expression were studied by western blotting. In addition, the levels of CD3 and CD28 proteins were measured by flow cytometry. In the current study, we found that CD28 deficiency significantly inhibited blast exposure-induced increases in the lung weight/body weight ratio and wet weight/dry weight ratio; decreased the infiltration of CD44^+^ leukocytes, CD163^+^ macrophages, and CD3^+^ T cells into the lungs; reduced the expressions of proinflammatory cytokines including IL-1*β*, TNF-*α*, and IL-6; and markedly increased IL-10 expression. CD28 deficiency also significantly attenuated blast exposure-induced ROS, MDA5, and IRE*α* expressions; increased SOD-1 expression; lowered the number of apoptotic cells and Bax, Caspase-3, and active Caspase-8 expressions; and increased Bcl-2 expression. Additionally, CD28 deficiency significantly ameliorated blast exposure-induced increases of p-PI3K and p-Akt and ameliorated the decrease in the p-FoxO1 expression. Our results suggest that CD28 deficiency has a protective effect on blast exposure-induced lung injury, which might be associated with the PI3K/Akt/FoxO1 signaling pathway.

## 1. Introduction

Blast exposure-induced injury is the most commonly encountered wounds in modern warfare. Traditionally associated with the battlefield environment, blast injuries are being increasingly observed among noncombatants because of increasing terrorist incidents, as well as gas and underground explosion events [1]. Blast injury is characterized by complex injuries, high shock rate, and high mortality and accounts for over 75% of all combat casualties in the United States forces [2]. An epidemiological analysis of the injuries in Operation Iraqi Freedom/Operation Enduring Freedom demonstrated that 81% of all injuries were associated with explosions [3]. A recent study showed that bomb blasts accounted for 82% of all injuries caused by terrorists and this number continues to rise [4]. The ears, lungs, and gastrointestinal tract are the most susceptible organs to blast injury, and lung injury is a major cause of high mortality. Sufficient energy exposure causes disruption of the capillary-alveolar interface, which leads to parenchymal hemorrhage and destruction of the alveolar walls. Interstitial changes in blast lungs gradually develop into acute respiratory distress syndrome and seriously affect the quality of life or prognosis. Therefore, it is of great importance to investigate the mechanism of blast exposure-induced lung injury for the treatment of wounded individuals.

Inflammatory responses are thought to play an important role in the development of blast exposure-induced lung injury, because increased lung leukocyte infiltration and elevated systemic and proinflammatory cytokine levels in the lungs are frequently associated with blast injury of the lung; furthermore, several factors including tumor necrosis factor-*α*, interleukin-6, and interleukin-1*β* accelerate lung tissue fibrosis and respiratory dysfunction [5]. In addition to inflammation, blast exposure also induced oxidative stress [6, 7] and apoptosis [8] in the lungs of experimental animals. Recent studies reported that T cell activation had a pivotal role in the development of inflammation [9, 10]. At least two signals are required for the full activation of T cells. Signal one requires the engagement of a T cell receptor by antigen-major histocompatibility complex proteins present on the surface of antigen-presenting cells [11], whereas signal two requires the engagement of CD28, a potent T cell costimulator expressed on antigen-presenting cells [12]. A blockade of CD28 signaling with antibodies or genetic ablation of CD28 in mice attenuated T cell activation. Barnett-Vanes et al. [13] found that blast exposure induced pulmonary barotrauma and inflammation, which were associated with increases of interleukin-6 (IL-6) and tumor necrosis factor-*α* (TNF-*α*). Bricker-Anthony et al. [14] demonstrated that blast exposure caused corneal edema and neovascularization, as well as immune cell infiltrate throughout the eyes. Struebing et al. [15] showed that ocular blast injury was related to the activation of the innate and acquired immune systems and infiltration of lymphocyte cells. Although it was demonstrated that CD28 signaling is associated with the expressions of inflammatory mediators, apoptosis-related proteins, immunosuppression, and tumorigenesis [16–19], the effects of CD28 deficiency on blast exposure-induced lung injury and activated T cell accumulation have not been investigated.

To demonstrate the mechanism of CD28 deficiency in blast exposure-induced lung injury, we tested the hypothesis that blast exposure-induced lung inflammation, oxidative stress, and apoptosis are attenuated in CD28 knockout (CD28^−/−^) mice. Our results suggest that CD28 deficiency significantly ameliorates blast exposure-induced lung inflammation, oxidative stress, and apoptosis and that CD28 deficiency is also associated with a reduction of T cell accumulation in blast exposure-induced lungs, which might be associated with the PI3K/Akt/FoxO1 signaling pathway. These findings suggest that CD28 deficiency is a promising therapeutic approach to mitigate blast exposure-induced lung injury.

## 2. Materials and Methods

### 2.1. Animals and Experimental Protocols

Thirty male CD28 knockout (CD28^−/−^) mice were obtained from the Jackson Laboratory, and thirty male C57BL/6 wild-type (WT) mice were provided by the Experimental Animal Department of the General Hospital of Northern Theater Command. All mice were kept in a room, maintained at a temperature of 20 ± 2°C and humidity of 55%–65%, and allowed free access to food and water in their cages. After acclimation, C57BL/6 WT and CD28^−/−^ mice were randomly divided into control or/and blast injury groups (model group). The control group was subdivided into two subgroups: C57BL/6 WT and CD28^−/−^ control groups. The model group was randomly divided into four groups: C57BL/6 24 h and 48 h and CD28^−/−^ 24 h and 48 h groups. Animal welfare and experimental design were approved by the Ethics Committee of the General Hospital of Northern Theater Command.

### 2.2. Establishment of Blast Injury Models

A model that mimics blast exposure-induced acute lung injury was used as previously described [20]. Briefly, mice were anesthetized by the abdominal injection of 2% pentobarbital sodium (1.5 ml/kg). The anesthetized mice were placed in a protective cover with their chest exposed, and aluminum foil was placed in the middle layer. After fixing the screws, the mouse was placed on the wire mesh atop the device. A pressure pump was used to increase the air pressure in the lower part until the aluminum foil burst. The compressed air rapidly expanded from the blasting port at high speed, forming shock waves that impacted the chest of the mouse. The pressure detected by a pressure sensor was transmitted through a data cable and recorded by a computer. Pressure waveform was obtained by the following formula: pressure (PSI) = voltage value∗1000/50.08. The instantaneous shock wave overpressure was 321 ± 24 PSI in this experiment.

### 2.3. Sample Collection and Processing

At 24 h and 48 h postblast, lung tissue samples were collected. Briefly, after 12 hours of fasting and 4 hours of water deprivation preoperatively, mice were intraperitoneally anesthetized with 2% sodium pentobarbital (1.5 ml/kg) and fixed in a prone position on the operating table. The abdominal cavity was opened, and blood was harvested through the abdominal aorta. The left lung was immersed in 10% formalin buffer for histological analysis, and the upper lobe of the right lung was dried with filter paper and weighed on an analytical balance to obtain the wet weight. After drying in an oven at 60°C for 72 hours, the dry weight of the right lung was recorded. The calculated ratio of dry weight to wet weight was an indicator of edema formation. The remaining lung was placed in a nitrogen canister for protein determination.

### 2.4. Histological Analysis

The left lung was fixed in 10% formaldehyde at room temperature and embedded in paraffin blocks using a Leica Microsystem tissue processor (ASP 300S, Germany) for histological analysis. For histological staining, sections of 3 *μ*m thickness were sliced using a Leica Microsystem microtome (Model RM 2265, Germany) and stained with hematoxylin and eosin (H&E).

### 2.5. Immunohistochemistry Staining

Sections were treated with 1% H_2_O_2_ to block endogenous peroxidase and then heated to 97°C in antigen retrieval solution for 15 min. The sample was incubated at room temperature for 60 min with mouse anti-rabbit CD44 (1 : 400, ab157107, Abcam, UK) and anti-rabbit CD163 antibodies (1 : 500, ab182422, Abcam, UK) and rinsed with phosphate buffer solution (PBS). After incubation with primary antibodies, the sections were washed with PBS and incubated with a secondary antibody (Sigma, USA), and then immunohistochemistry visualization was completed using 3,3-diaminobenzidine (DAB) (Sigma, USA) and counterstained with hematoxylin solution. A negative control consisted of the same immunohistochemical method with the omission of the primary antibodies. Cells with yellow and brown particles in their cytoplasm and/or nuclei were identified as positive cells.

### 2.6. Reactive Oxygen Species (ROS) Detection

Frozen sections of the right lobe of the lungs were immersed in PBS twice for 5 minutes each time, incubated with 10 *μ*M dihydroethidium in the dark for 20 minutes, immersed again in PBS twice for 5 minutes each time, and observed and photographed by a fluorescence microscope.

### 2.7. TUNEL Staining

Lung tissue apoptosis was detected in accordance with the instructions of the TUNEL kit (Cloud-Clone Company, USA). The nuclei of apoptotic cells were stained brown. Five different fields were randomly selected under a high-power microscope (×400) to calculate the apoptotic index.

### 2.8. Immunofluorescence Staining

Immunofluorescence staining was performed to localize p-PI3K (1 : 200, #4228S, Cell Signaling Technology, USA), p-Akt (1 : 400, #4051S, Cell Signaling Technology, USA), and p-FoxO1 (1 : 250, ab131339, Abcam, UK). Lung tissues were dewaxed with xylene, hydrated with a graded alcohol series, incubated with 0.1% Triton X-100 for 30 minutes, and washed three times with PBS for 5 minutes each time. Samples were blocked with 5% bovine serum albumin and 10% goat serum, each for 30 minutes, incubated with a primary antibody overnight at 4°C in a wet box, and stained with a fluorescent secondary antibody. Finally, samples were observed and photographed with a microscope.

### 2.9. Western Blotting

Western blotting was performed as described previously [21]. Whole protein was extracted from the lung tissues using a tissue protein extraction kit (Cat No: FD0889 (year 2016), Hangzhou Fude Biological Technology Company, China), and the protein concentration was determined using a BCA protein assay kit (Cat No: FD2001 (year 2017), Hangzhou Fude Biological Technology Company, China). Equal amounts of soluble protein were separated on 10% polyacrylamide gels, transferred onto a nitrocellulose membrane, and followed by routine western blot analysis. The primary antibody is composed of the following: TNF-*α* (1 : 2000, ab8348, Abcam, UK), IL-10 (1 : 2000, ab9969, Abcam, UK), IL-6 (1 : 1000, ab83053, Abcam, UK), IRE*α* (1 : 2000, #3294, Abcam, UK), and MDA5 (1 : 200, ab69983, Abcam, UK). IL-1*β* (1 : 2000, sc-7884, Santa Cruz Biotechnology Inc., USA), IL-4 (1 : 1000, sc-73318, Santa Cruz Biotechnology Inc., USA), SOD-1 (1 : 1000, sc-11407, Santa Cruz Biotechnology Inc., USA), Bax (1 : 2000, sc-526, Santa Cruz Biotechnology Inc., USA), Caspase-3 (1 : 500, sc-7148, Santa Cruz Biotechnology Inc., USA), Bcl-2 (1 : 1000, sc-7382, Santa Cruz Biotechnology Inc., USA), active Caspase-8 (1 : 100, sc-5263, Santa Cruz Biotechnology Inc., USA), PI3K (1 : 1000, ab186612, Abcam, UK), p-PI3K (1 : 1000, #4228S, Cell Signaling Technology, USA), Act (1 : 2000, ab32505, Abcam, USA), p-Act (1 : 2000, #4051S, Cell Signaling Technology, USA), Forkhead box O1 (FoxO1) (1 : 1000, #2880, Cell Signaling Technology, USA), p-FoxO1 (1 : 1000, ab131339, Abcam, UK), and GAPDH (1 : 5000, sc-32233, Santa Cruz Biotechnology Inc., USA) were obtained. The secondary antibody was goat anti-mouse secondary antibody (HRP) (1 : 4000 mouse IgG, ab6789, Abcam, UK), goat anti-rabbit secondary antibody (HRP) (1 : 4000, ab6721, Abcam, UK), and goat anti-rat secondary antibody (HRP) (1 : 2000, ab7097, Abcam, UK). Proteins were visualized using a Clarity™ Western ECL Substrate (#170-5061; Bio-Rad Laboratories Inc., USA) and a Tanon 5200 Full automatic chemiluminescence image analysis system (Tanon Science and Technology Co. Ltd., Shanghai, China).

### 2.10. Flow Cytometry

Flow cytometric analysis was performed as described previously [22]. Briefly, individual lungs were excised, cut into small pieces, and enzymatically digested in 5 ml of digestion buffer (HBSS without Ca+/Mg+ (Life Technologies) and 1 mg/ml collagenase (Roche)) at 37°C for 30 min with agitation and then pressed against the bottom of a 100 *μ*m strainer with the plunger of a 3 ml syringe. Single cells from tissues were washed through the strainer with 10 ml cold buffer (PBS + 0.5%BSA + 2 mM EDTA). After erythrocyte lysis using Red Blood Cell Lysing Buffer (Sigma), cells were counted using a hemocytometer. Single cell suspensions were preincubated with anti-mouse CD3 or CD28 antibodies (BD Biosciences, Franklin Lakes, NJ, USA) to prevent nonspecific binding of antibodies to FcR*γ*, followed by multistaining with fluorescence conjugated primary antibodies. Dead cells were stained with propidium iodide staining solution. Samples were subjected to FACS Aria II analysis (BD Biosciences). Data were analyzed by FlowJo software.

### 2.11. Statistical Analysis

Data are expressed as the mean ± standard deviation and were analyzed using SPSS 20.0 statistical software. All experiments were repeated at least 3 times. Significance was determined when *P* values < 0.05 were obtained by two-way ANOVA with the Bonferroni post hoc test.

## 3. Results

### 3.1. CD28 Deficiency Attenuates Blast Exposure-Induced Acute Lung Injury

**Figure 1:**
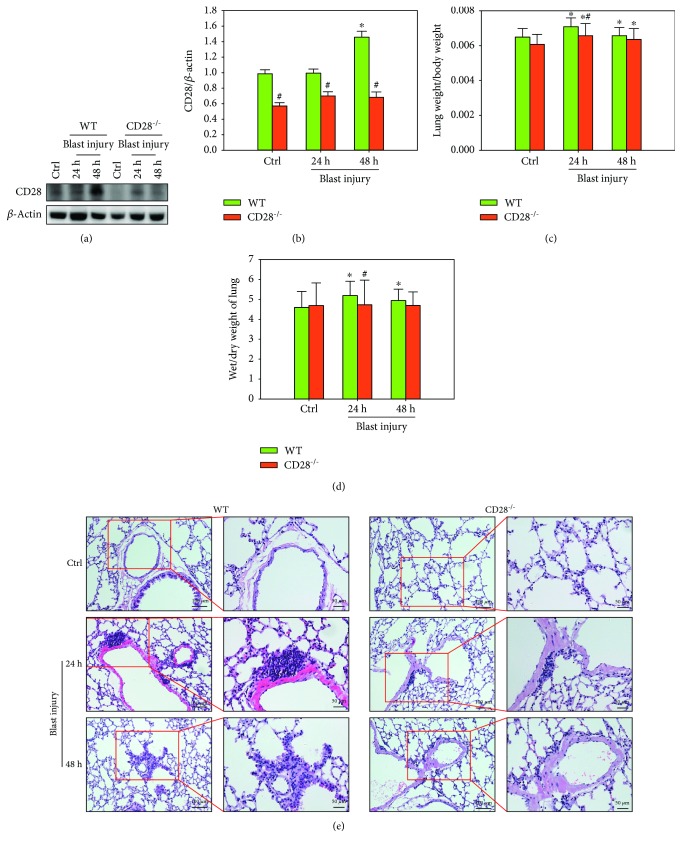
CD28 deficiency attenuates blast exposure-induced acute lung injury. (a, b) Representative western blot images and quantitative analysis of CD28 and *β*-actin. Fold changes in WT (green bar) and CD28^−/−^ (red bar) expressions are shown. (c, d) Lung weight/body weight and the ratio of wet weight to dry weight in each group. (e) Representative histopathological images of each group (HE staining, scale bar = 50 *μ*m). All experiments were repeated at least three times. Results are expressed as the mean ± SEM (*n* = 10 per group). ^∗^*P* < 0.05 versus the control group; ^#^*P* < 0.05 versus the WT group (two-way ANOVA; Bonferroni post hoc test). WT: C57BL/6 wild-type mice; CD28^−/−^: CD28 knockout mice.

Compared with the control group, the level of CD28 protein was significantly elevated in the model group, whereas CD28 deficiency reduced blast exposure-induced levels of CD28 compared with the C57BL/6 model group (Figures 1(a) and 1(b), *P* < 0.05). Blast exposure significantly increased the ratio of lung weight/body weight and the ratio of wet weight/dry weight compared with the control group, whereas CD28 deficiency significantly inhibited blast exposure-induced increases of the ratios compared with the C57BL/6 model group (Figures 1(c) and 1(d), *P* < 0.05). In addition, serious lung hemorrhage, edema, and obvious inflammatory cell infiltration were observed in the C57BL/6 model group, whereas these were attenuated in the CD28^−/−^ model group (Figure 1(e)). These data demonstrate that CD28 deficiency was effective at ameliorating blast exposure-induced acute lung injury.

### 3.2. CD28 Deficiency Inhibits Blast Exposure-Induced Lung Proinflammatory Cytokine Expression

**Figure 2:**
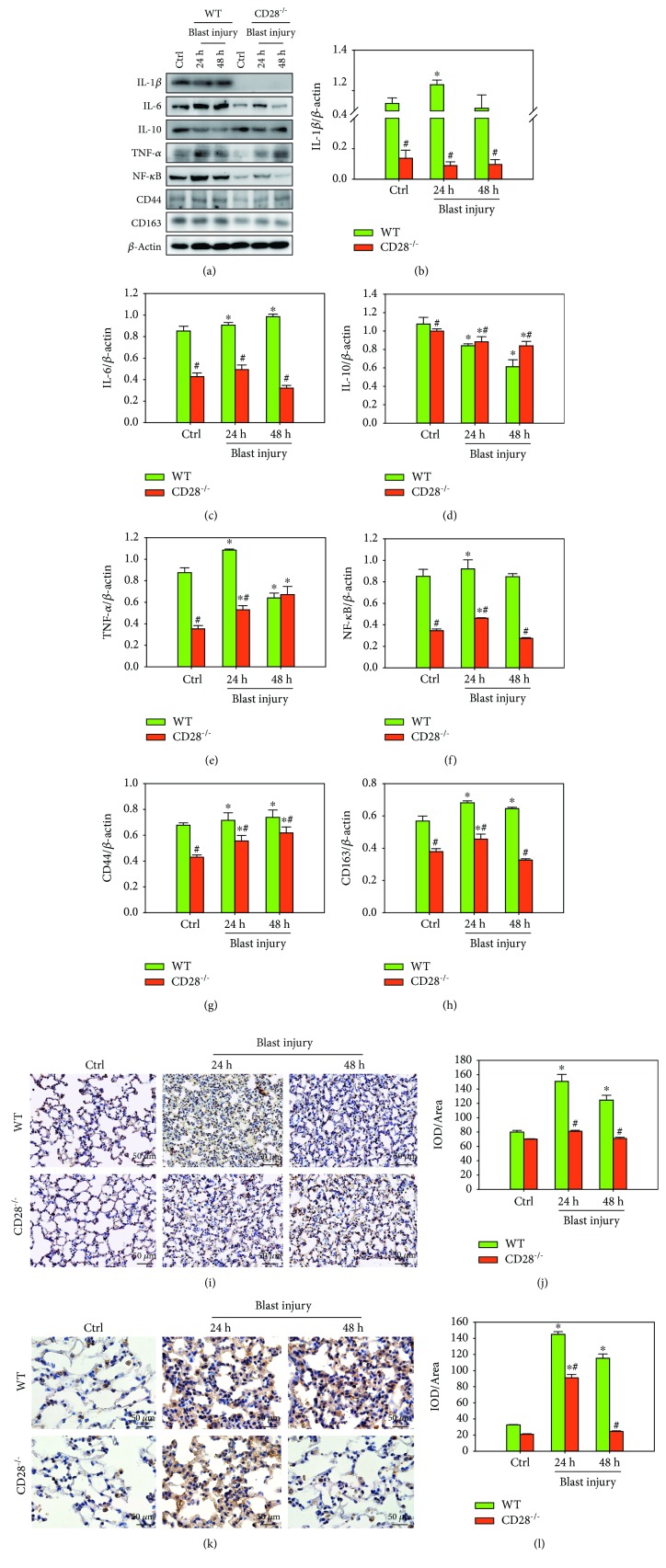
CD28 deficiency inhibits blast exposure-induced lung proinflammatory cytokine expression. (a–h) Representative western blot images and quantitative analysis of IL-1*β*, IL-6, IL-10, TNF-*α*, NF-*κ*B, CD44, CD163, and *β*-actin. Fold changes of WT (green bar) and CD28^−/−^ (red bar) expressions are shown. (i–l) Representative immunohistochemical images and semiquantitative analysis of CD44 and CD163 protein in each group (immunohistochemical staining, scale bar = 50 *μ*m). All experiments were repeated at least three times. Results are expressed as the mean ± SEM (*n* = 10 per group). ^∗^*P* < 0.05 versus the control group; ^#^*P* < 0.05 versus the WT group (two-way ANOVA; Bonferroni post hoc test). WT: C57BL/6 wild-type mice; CD28^−/−^: CD28 knockout mice.

Both CD28 deficiency and blast affected the expression of IL-1*β*, IL-6, TNF-*α*, NF-*κ*B, CD44, CD163, and IL-10. At 24 h and 48 h postblast, higher expressions of IL-1*β*, IL-6, TNF-*α*, NF-*κ*B, CD44, and CD163 and a lower expression of IL-10 were observed in the control group compared with the CD28^−/−^ model group (Figure 2(a)–2(h), *P* < 0.05). Moreover, immunohistochemical data also demonstrated that the expression levels of CD44- and CD163-positive inflammatory cells in the CD28^−/−^ model group were significantly lower than those in the C57BL/6 model group (Figure 2(i)–2(l), *P* < 0.05). These data demonstrate that CD28 deficiency was effective at ameliorating blast exposure-induced lung proinflammatory cytokine expression.

### 3.3. CD28 Deficiency Inhibits Blast Exposure-Induced Expression of ROS and Oxidative Stress

**Figure 3:**
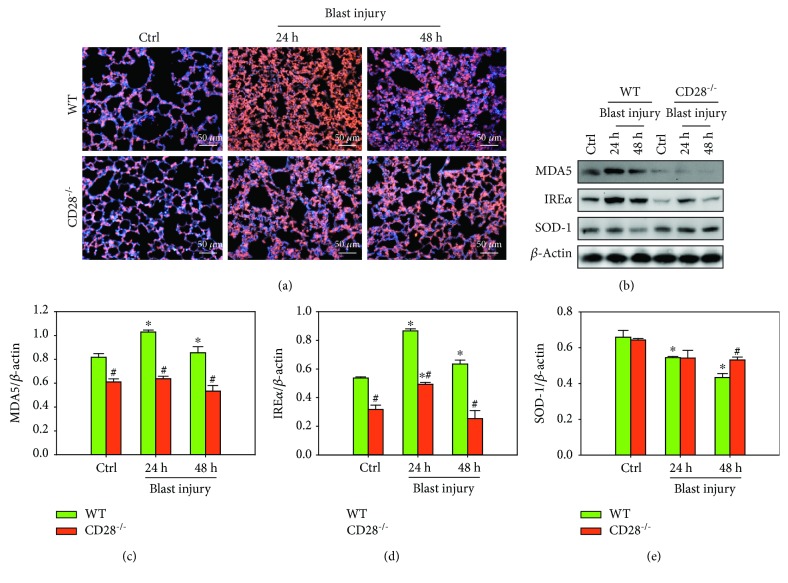
CD28 deficiency inhibits the blast exposure-induced expression of ROS and oxidative stress. (a) Representative ROS images from the lung tissues (scale bar = 50 *μ*m). (b–e) Representative western blot images and quantitative analysis of MDA5, IRE*α*, SOD-1, and *β*-actin. Fold changes of WT (green bar) and CD28^−/−^ (red bar) expressions are shown. All experiments were repeated at least three times. Results are expressed as the mean ± SEM (*n* = 10 per group). ^∗^*P* < 0.05 versus the control group; ^#^*P* < 0.05 versus the WT group (two-way ANOVA; Bonferroni post hoc test). WT: C57BL/6 wild-type mice; CD28^−/−^: CD28 knockout mice.

Compared with the control group, the expression of ROS was significantly higher in the model group, while CD28 deficiency significantly decreased blast-induced ROS expression compared with the C57BL/6 model group (Figure 3(a)). Western blot results showed that blast exposure increased the levels of MDA5 and IRE*α* proteins and decreased SOD-1 protein compared with the control group, whereas blast-induced increases of MDA5 and IRE*α* protein and decreases of SOD-1 protein were significantly attenuated by CD28 deficiency compared with the C57BL/6 model group (Figure 3(b)–3(e), *P* < 0.05). This demonstrated that CD28 deficiency is effective at ameliorating blast exposure-induced lung reactive oxygen species and oxidative stress.

### 3.4. CD28 Deficiency Ameliorates Blast Exposure-Induced Lung Tissue Apoptosis

**Figure 4:**
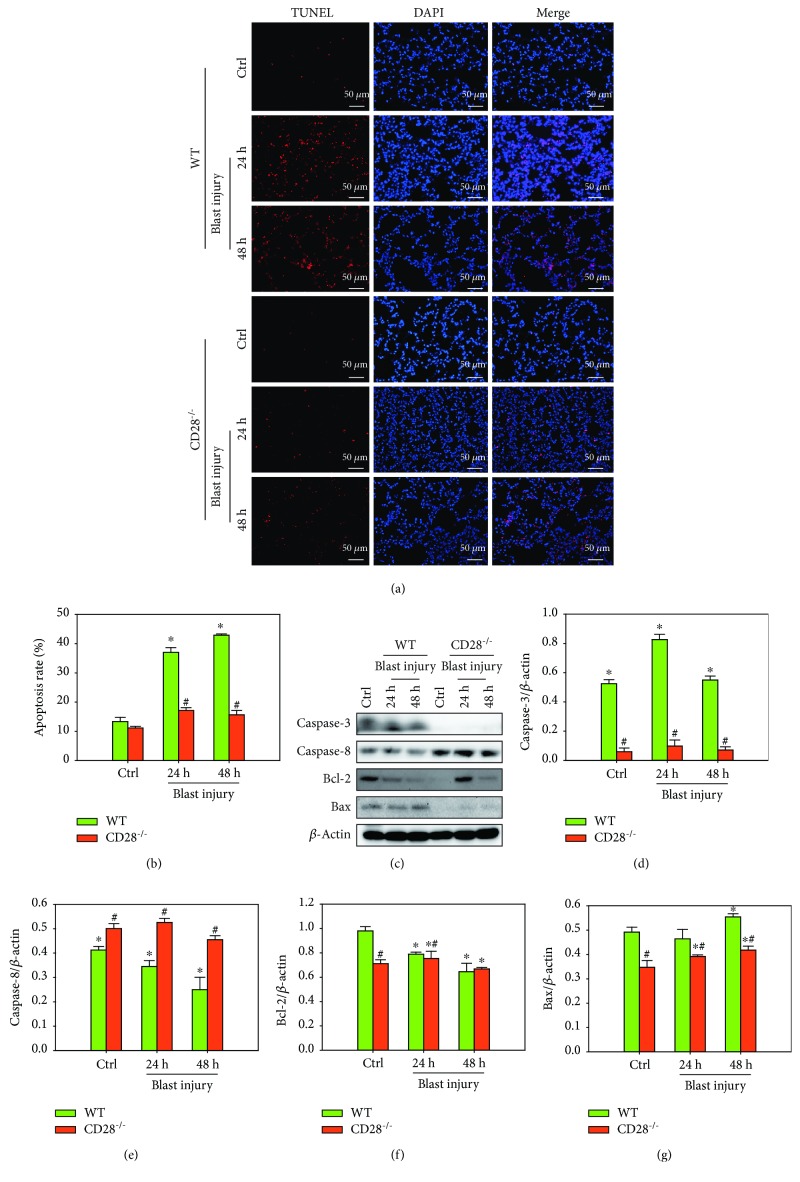
CD28 deficiency ameliorates blast exposure-induced lung tissue apoptosis. (a, b) Representative images and semiquantitative analysis of lung tissue apoptosis (TUNEL staining, scale bar = 50 *μ*m). (c–g) Representative western blot images and quantitative analysis of Caspase-3, Caspase-8, Bcl-2, Bax, and *β*-actin in each group. Fold changes of WT (green bar) and CD28^−/−^ (red bar) expressions are shown. All experiments were repeated at least three times. Results are expressed as the mean ± SEM (*n* = 10 per group). ^∗^*P* < 0.05 versus the control group; ^#^*P* < 0.05 versus the WT group (two-way ANOVA; Bonferroni post hoc test). WT: C57BL/6 wild-type mice; CD28^−/−^: CD28 knockout mice.

TUNEL results showed that the number of apoptotic cells in the model group was much higher than that in the control group and that the number of apoptotic cells induced by blast injury was significantly decreased by CD28 deficiency compared with the C57BL/6 model group (Figures 4(a) and 4(b), *P* < 0.05). These results demonstrated that blast exposure increased the levels of the proapoptotic proteins Bax, Caspase-3, and active Caspase-8 and decreased the levels of the antiapoptotic proteins Bcl-2 compared with the control group, whereas CD28 deficiency evidently attenuated blast exposure-induced increases of Bax, Caspase-3, and active Caspase-8 and decreased Bcl-2 compared with the C57BL/6 model group (Figure 4(b)–4(g), *P* < 0.05). These data suggest that CD28 deficiency is effective at ameliorating blast exposure-induced lung tissue apoptosis.

### 3.5. CD28 Deficiency Inhibits CD3^+^ T Cell Accumulation in Blast Exposure-Induced Lung Tissues

**Figure 5:**
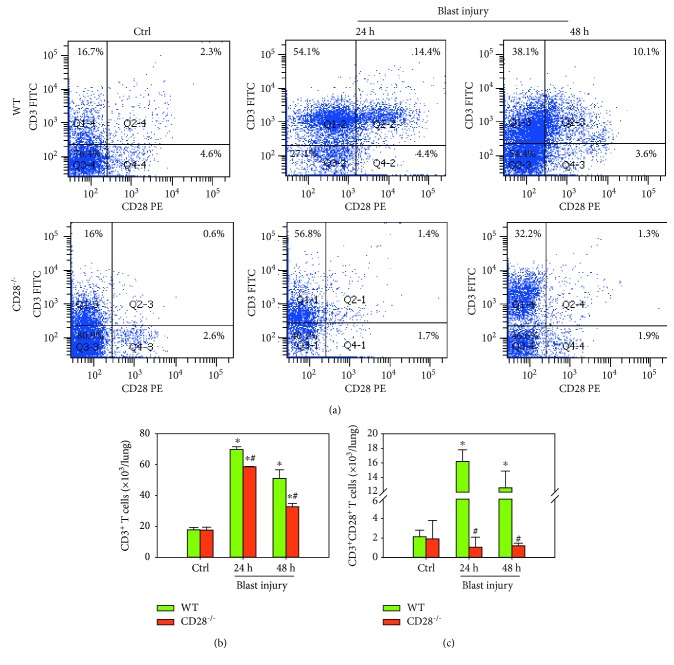
CD28 deficiency inhibits CD3^+^ T cell accumulation in blast exposure-induced lung tissues. At 24 h and 48 h postblast, flow cytometry data were collected from C57BL/6 WT and CD28^−/−^ mice. (a, b) Flow cytometry plots and quantitative data represent the percentage of CD3^+^ T cells in lungs. (c) Quantitative data represent the total numbers of CD3^+^ T cells and CD3^+^ CD28^+^ T cells in lungs after blast injury. All experiments were repeated at least three times. Results are expressed as the mean ± SEM (*n* = 10 per group). ^∗^*P* < 0.05 versus the control group; ^#^*P* < 0.05 versus the WT group (two-way ANOVA; Bonferroni post hoc test). WT: C57BL/6 wild-type mice; CD28^−/−^: CD28 knockout mice.

At 24 h and 48 h postblast, the percentage of total CD3^+^ T cells in lungs was remarkably increased compared with the control group, while it was significantly ameliorated by CD28 deficiency, which decreased the accumulation of total CD3^+^ T cells and inhibited early activated T cells compared to the C57BL/6 model group (Figure 5, *P* < 0.05). These results show that CD28 deficiency is effective in ameliorating blast exposure-induced CD3^+^ T cell accumulation in lungs.

### 3.6. CD28 Deficiency Attenuates Blast Exposure-Induced Lung Injury through the PI3K/Akt/FoxO1 Signaling Pathway

**Figure 6:**
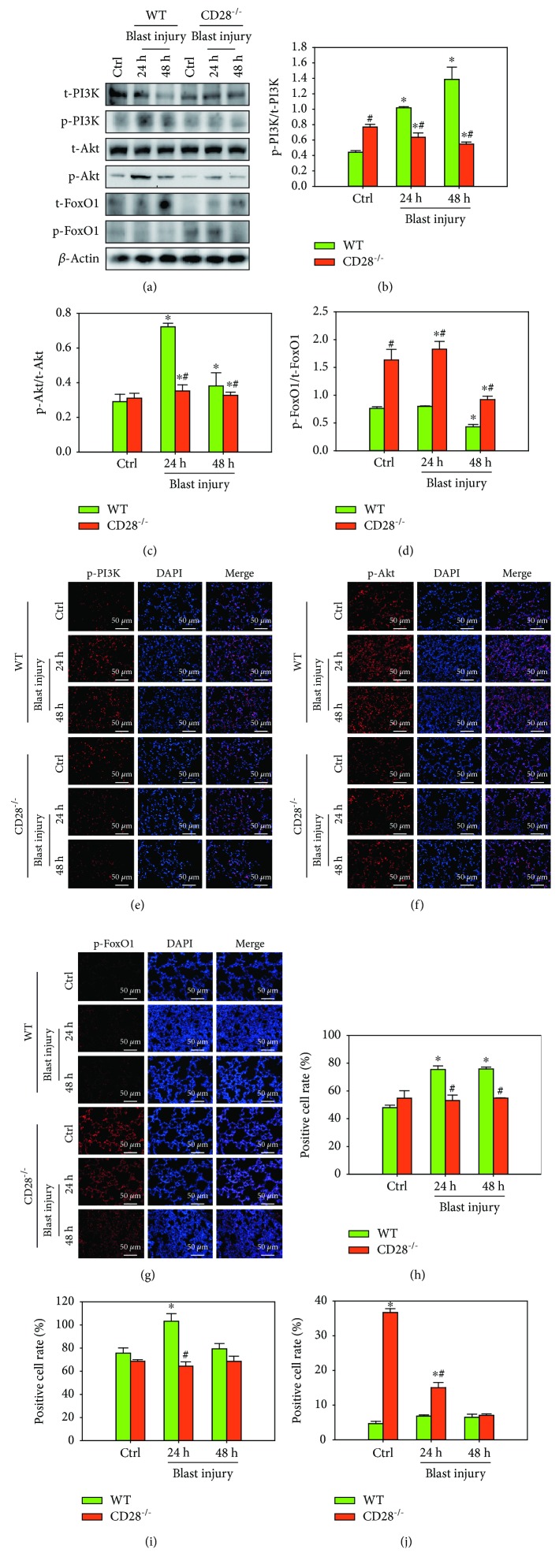
CD28 deficiency attenuates blast exposure-induced lung injury through the PI3K/Akt/FoxO1 signaling pathway. (a) Representative western blot images of t-PI3K, p-PI3K, t-Akt, p-Akt, t-FoxO1, p-FoxO1, and *β*-actin. (b–d) Quantitative analysis of each group was conducted. Fold changes of WT (green bar) and CD28^−/−^ (red bar) expressions are shown. (e–g) Representative immunofluorescent staining images of p-PI3K, p-Akt, and p-FoxO1 in the lungs. (h–j) Quantitative analysis of each group was performed. Fold changes of WT (green bar) and CD28^−/−^ (red bar) expressions are shown. All experiments were repeated at least three times. Results are expressed as the mean ± SEM (*n* = 10 per group). ^∗^*P* < 0.05 versus the control group; ^#^*P* < 0.05 versus the WT group (two-way ANOVA; Bonferroni post hoc test). WT: C57BL/6 wild-type mice; CD28^−/−^: CD28 knockout mice.

The expression levels of p-PI3K, p-Akt, and p-FoxO1 proteins were not altered in CD28^−/−^ mice under control conditions. At 24 h and 48 h postblast, western blot results showed that the expressions of p-PI3K and p-Akt were significantly increased and p-FoxO1 levels were significantly decreased compared with the control group, whereas these factors were significantly attenuated by CD28 deficiency as compared with the C57BL/6 model group (Figure 6(a)–6(d), *P* < 0.05). Moreover, immunofluorescence results also confirmed that blast exposure induced the higher expressions of p-PI3K and p-Akt and lower expression of p-FoxO1 in the CD28^−/−^ model group compared with the C57BL/6 model group (Figure 6(e)–6(j)). These data demonstrate that CD28 deficiency is effective at ameliorating blast exposure-induced lung injury via the PI3K/Akt/FoxO1 signaling pathway.

## 4. Discussion

Blast exposure induces injury in several organs including the lungs [23], brain [24, 25], heart [26], ears [27], jejunum [28], and kidney [29]. A number of studies demonstrated that blast exposure caused inflammation, oxidative stress, and cell apoptosis in the lungs of experimental animals and humans [20, 30, 31]. In this study, we showed that CD28 deficiency ameliorated blast exposure-induced acute lung injury, reduced proinflammatory cytokine expression, decreased oxidative stress and cell apoptosis, and also inhibited CD3^+^ T cell accumulation in lung tissues. Additionally, CD28 deficiency significantly ameliorated blast exposure-induced increases in p-PI3K and p-Akt expressions and ameliorated decreases in p-FoxO1 expression. Therefore, we propose that the attenuation of blast exposure-induced lung injury by CD28 deficiency might be associated with the activation of the PI3K/Akt/FoxO1 signaling pathway.

In this study, our results demonstrated that CD28 deficiency ameliorated blast exposure-induced acute lung injury and inhibited proinflammatory cytokine expression. Several studies demonstrated that CD28-mediated signaling prevented the spontaneous development of autoimmune diabetes [32] and decreased experimental autoimmune neuritis [33] and CD28 deficiency was highly resistant to collagen-induced arthritis in mice [34]. Similar to our results, Macal and Tam [35] reported that the levels of the proinflammatory cytokines TNF-*α* and IL-6 were significantly decreased in CD28-deficient pDCs compared with WT pDCs. Moreover, *in vivo* CD28 knockdown decreased the levels of COX-2, IL-10, and IL-17A mRNAs at day 24 in a chronic UVB exposure study [16]. Whereas the CD28-B7 blockade by CTLA4-Ig inhibited IFN-*γ* production in C57BL/6 recipients, it had little effect on the production of IL-6 [36]. Similarly, the administration of a CD28 antagonist peptide decreased irradiation-induced increase in IL-6 and lowered COX-2 expression and the numbers of macrophages in the small intestine of irradiated mice [37]. The blockade of CD28 signals also exacerbated left ventricular remodeling and increased cardiac rupture after myocardial infarction by prolonging the inflammatory period, which caused a reduction in collagen fibers in infarct scars [38]. These results suggest that CD28 deficiency is effective at ameliorating blast exposure-induced lung inflammation.

In addition to its anti-inflammatory effects, we also showed that CD28 deficiency decreased blast exposure-induced oxidative stress and cell apoptosis. It was reported that deltamethrin induced apoptosis through its interaction with CD28 receptors, leading to oxidative stress and activation of the mitochondrial caspase-dependent pathways, which finally affected the functions of the immune system [39]. In addition, the CTLA4-Ig blockade of synovial adherent cell signaling to CD28 T cells prevented ROS induction in T lymphocytes exposed to inflammatory cytokines [40]. Similar results were reported by Ma et al. [41] where the blockade of the CD80/CD28 signaling pathway promoted recovery of spatial memory, which was associated with mechanisms involving the CD80/CD28 pathway that regulates inflammatory and immune responses and downregulates programmed cell death through apoptosis and autophagy. Our results also showed that CD28 deficiency decreased blast exposure-induced T cell accumulation. Similarly, Suresh et al. [42] demonstrated that the CD28 signaling pathway regulated the generation and maintenance of the immune responses. Soluble CD28 promoted T cell responses, leading to T cell activation and proliferation [43]. Zaitsu et al. [44] reported that the blockade of selective CD28 promoted Treg functions and the regulation of alloimmune responses *in vivo*. Moreover, Wang et al. [22] showed that the CD28/B7 blockade by CTLA4-Ig treatment and CD28/B7 KO inhibited activated effector T cell accumulation in transverse aortic constriction-induced heart failure. These studies indicate that CD28 deficiency might have a protective role against blast exposure-induced lung injury by ameliorating oxidative stress, apoptosis, and activated T cell accumulation.

**Figure 7:**
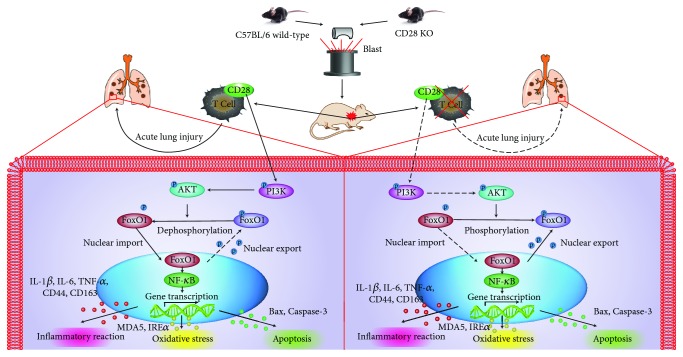
Schematic representation of CD28 KO reverses lung blast injury by the PI3K/Akt/FoxO1 signaling pathway. Blast exposure induces the phosphorylation of the PI3K protein, which activates the Akt protein in C57BL/6 mice. Once Akt is activated, it induces p-FoxO1, a downstream target protein of Akt, which is dephosphorylated to form FoxO1 that is transferred to the nucleus. Therefore, we hypothesize that FoxO1 upregulates the expressions of IL-1*β*, IL-6, TNF-*α*, NF-*κ*B, CD44, active Caspase-8, CD163, MDA5, IRE*α*, Bax, and Caspase-3 expression and downregulates the expressions of IL-10, SOD-1, and Bcl-2, which regulates inflammation, oxidative stress, and apoptosis, while CD28 KO might reduce PI3K/Akt phosphorylation and increase FoxO1 phosphorylation, which reverses blast exposure-induced lung injury. WT: C57BL/6 wild-type mice; CD28^−/−^: CD28 knockout mice.

Our results showed that CD28 deficiency decreased blast exposure-induced lung injury, which might be associated with the PI3K/Akt/FoxO1 signaling pathway. It was recently reported that CD28 signaling in T cells is related closely to the phosphatidylinositol 3-kinase (PI3K)/Akt pathway [45, 46]. Similar to T cells, CD28 activation in multiple myeloma cells activated PI3K/Akt signaling, which might regulate the apoptotic resistance of multiple myeloma [47, 48]. FoxO1 knockdown partly prevented the blockade of CD28 and led to death, whereas the activation of CD28 could increase p-FoxO1 [49]. A recent study showed that the PI3K/Akt pathway was required for the CD28-induced activation of NF-*κ*B and the CD28-induced signaling pathway, which lead to significantly increased of T cell proliferation and cytokine production [50]. Furthermore, blocking CD28 inhibited chemotherapy-induced cell apoptosis, and the CD28 signaling pathway was associated with the downstream activation of PI3K/Akt and inactivation of the FoxO1 in multiple myeloma [51]. Based on the above results, we speculated on the putative molecular mechanism involved in the CD28 regulation of blast injury (Figure 7). Blast exposure induces the phosphorylation of PI3K protein, which activates Akt proteins in C57BL/6 mice. Once Akt is activated, it induces p-FoxO1, a downstream target protein of Akt, which is then dephosphorylated to form FoxO1 and transferred to the nucleus. Therefore, we hypothesize that FoxO1 upregulates IL-1*β*, IL-6, TNF-*α*, NF-*κ*B, CD44, active Caspase-8, CD163, MDA5, IRE*α*, Bax, and Caspase-3 expressions; downregulates the expressions of IL-10, SOD-1, and Bcl-2; and then regulates inflammation, oxidative stress, and apoptosis. CD28 KO may reduce PI3K/Akt phosphorylation and increase FoxO1 phosphorylation, which finally reverses blast exposure-induced lung injury. More experiments are required to further study and clarify the detailed mechanism of this phenomenon.

In summary, blast exposure leads to inflammation, oxidative stress, and apoptosis in mouse lungs. CD28 deficiency has a protective effect on blast exposure-induced lung injury, which might be associated with the PI3K/Akt/FoxO1 signaling pathway.

## Data Availability

All authors declare that all data are fully available without restriction.
